# Carcinosarcoma Arising From the Renal Pelvis: A Rare Case Report

**DOI:** 10.7759/cureus.45610

**Published:** 2023-09-20

**Authors:** Taner Hacıosmanoğlu, İbrahim H Baloğlu, Semih Türk, Hüseyin C Demirel, Ayşe A Özağarı

**Affiliations:** 1 Department of Urology, Sisli Hamidiye Etfal Training and Research Hospital, University of Health Sciences, Istanbul, TUR; 2 Department of Urology, Sultan 1. Murat State Hospital, Edirne, TUR; 3 Department of Urology, Istanbul Aydin University VM Medical Park Florya Hospital, Istanbul, TUR; 4 Department of Pathology, Sisli Hamidiye Etfal Training and Research Hospital, University of Health Sciences, Istanbul, TUR

**Keywords:** kidney stone disease, nephrocutaneous fistula, radical nephrectomy, renal pelvis tumor, carcinosarcoma

## Abstract

Carcinosarcoma is a malignant tumor of biphasic character consisting of epithelial and mesenchymal components. This rarely-seen tumor has an exceedingly aggressive nature. While it is rare in the urinary system, it appears even more rarely in the renal pelvis. Thus, there are few publications in the literature on carcinosarcomas originating from the renal pelvis. This paper presents a 42-year-old male patient with carcinosarcoma of the renal pelvis (CSRP), kidney stone disease, and a nephrocutaneous fistula who underwent radical nephrectomy and eventually died of metastatic disease. The rarity of the disease is the main obstacle to conducting comprehensive clinical trials. Therefore, it is of great importance to publish the identified carcinosarcoma of the renal pelvis cases.

## Introduction

Carcinosarcoma is a malignant tumor of a biphasic character comprising epithelial and mesenchymal components [1]. It is rarely seen and has an exceedingly aggressive nature [2]. Squamous cell carcinoma (SCC), adenocarcinoma, or transitional cell carcinoma (TCC) may be observed as the carcinomatous component, while chondrosarcoma, osteosarcoma, rhabdomyosarcoma, liposarcoma, or fibrosarcoma may be observed as the sarcomatous component [3]. Carcinosarcoma is most commonly seen in the uterus, breast, esophagus, lungs, and prostate gland [4]. It is rare in the urinary system and appears even more rarely in the renal pelvis [4]. Thus, there are few publications in the literature on carcinosarcomas originating from the renal pelvis, and limited data are available on this disease. This paper presents a patient with a carcinosarcoma of the renal pelvis (CSRP), kidney stone disease, and a nephrocutaneous fistula.

## Case presentation

**Figure 1:**
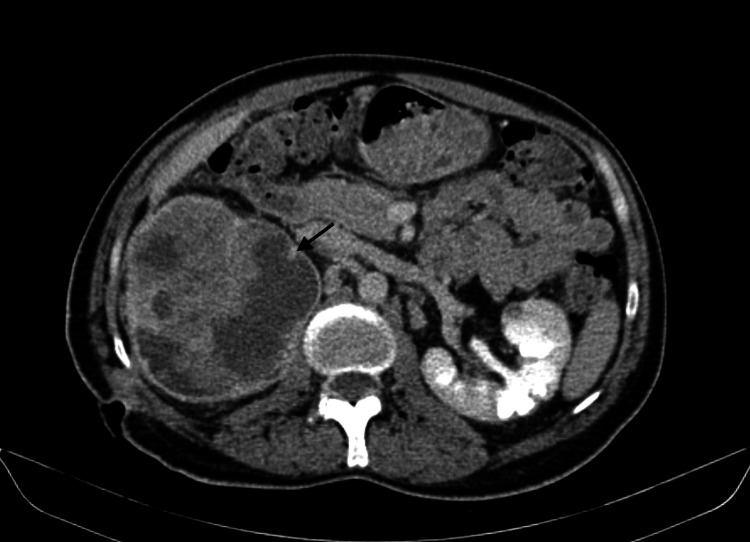
An intravenous contrast-enhanced abdominal CT section showing a giant cystic mass with solid components and heterogeneous contrast uptake with an arrow. The edges of the mass are well-defined, it has a capsule-like contrast enhancement, and the nephrocutaneous fistula is seen.

A 42-year-old male patient with a known nephrolithiasis history was referred to our outpatient urology clinic with a complaint of right flank pain and smelly discharge from the right flank area for about one year. He had no history of cancer, comorbidity, or allergy. There was no operation in the patient's history except for the right percutaneous nephrolithotomy (PCNL), which he had 1.5 years ago due to staghorn calculi. At that time, the patient's examinations did not reveal any findings in favor of a kidney tumor. He had been smoking an average of two packs of cigarettes per day for 17 years. On physical examination, there was a fistula in the right lumbar region from which foul-smelling urine came out, and there were no other abnormal findings. The laboratory analysis showed that the patient's serum hemoglobin (Hb) was 9.8 g/dL, his white blood cell (WBC) count was 14 × 109 leukocytes/L, and his C-reactive protein (CRP) was 60 mg/L. His kidney function tests, liver function tests, serum corrected calcium levels, serum alkaline phosphatase levels, and lactate dehydrogenase levels were all normal. The urine culture indicated a urinary infection. A non-contrast abdominal computed tomography (CT) was performed, and it showed the fistula tract between the right kidney and the skin. Moreover, there was a giant incidental mass-like lesion in the right kidney in addition to the rest of the renal stones after PCNL. An intravenous contrast-enhanced abdominal CT was then performed and showed a giant cystic mass with solid components, 13 cm × 11 cm in size, which had heterogeneous contrast uptake (Figure 1).

**Figure 2:**
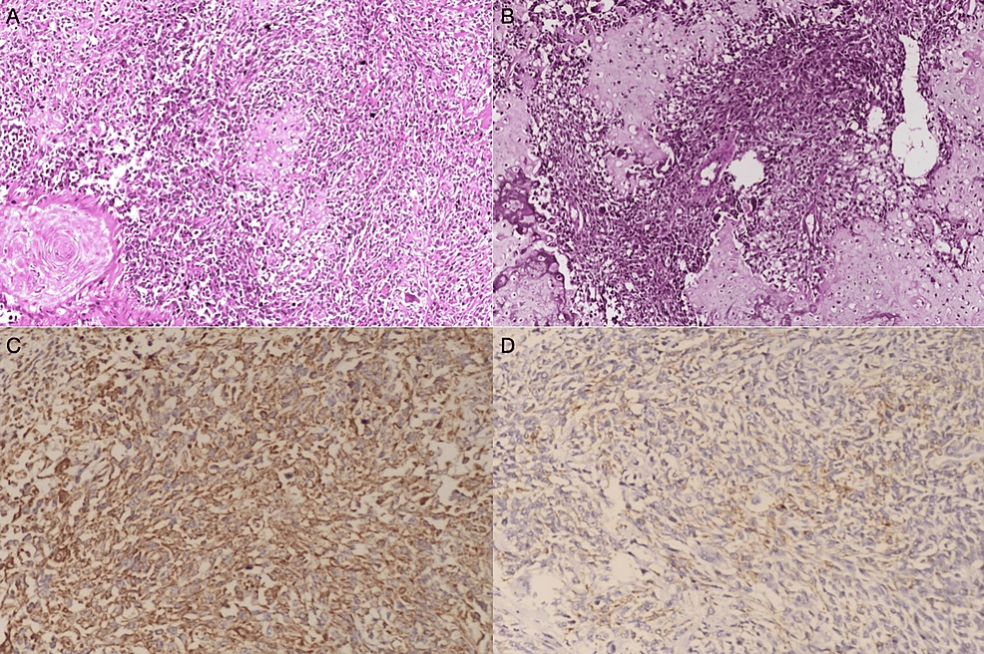
Carcinosarcoma (sarcomatoid carcinoma) showing both malignant epithelial (SCC) and malignant spindle cell components (hematoxylin&eosin ×20) (A). Tumor areas showing chondrosarcomatous component (hematoxylin and eosin ×20) (B). Immunohistochemical vimentin (C) and CK (D) positivity in spindle cell areas are shown here (×40).

Liver involvement was not noted in this imaging, and no finding in favor of venous involvement was found. A contrast-enhanced thoracic CT was performed, and there was no lung metastasis. Additionally, lymph node metastasis was not observed in the CTs. Based on these findings, we planned radical nephrectomy and fistulectomy after the patient was administered antibiotics due to the urinary infection. After antibiotic treatment, the patient's WBC count and CRP level decreased to normal levels, and the patient was operated on. During the operation, findings suggesting that the tumor was attached to the liver were observed, and excision of the margins of the liver was performed. Additionally, lymph nodes in the hilar region, two of which were palpable, were excised. One week after surgery, the patient was discharged without any complications. The pathological analysis of the nephrectomy specimen showed carcinosarcoma originating from the renal pelvis (Figure 2), and findings in support of the tumor were observed on the inner end of the fistulectomy material.

**Figure 3:**
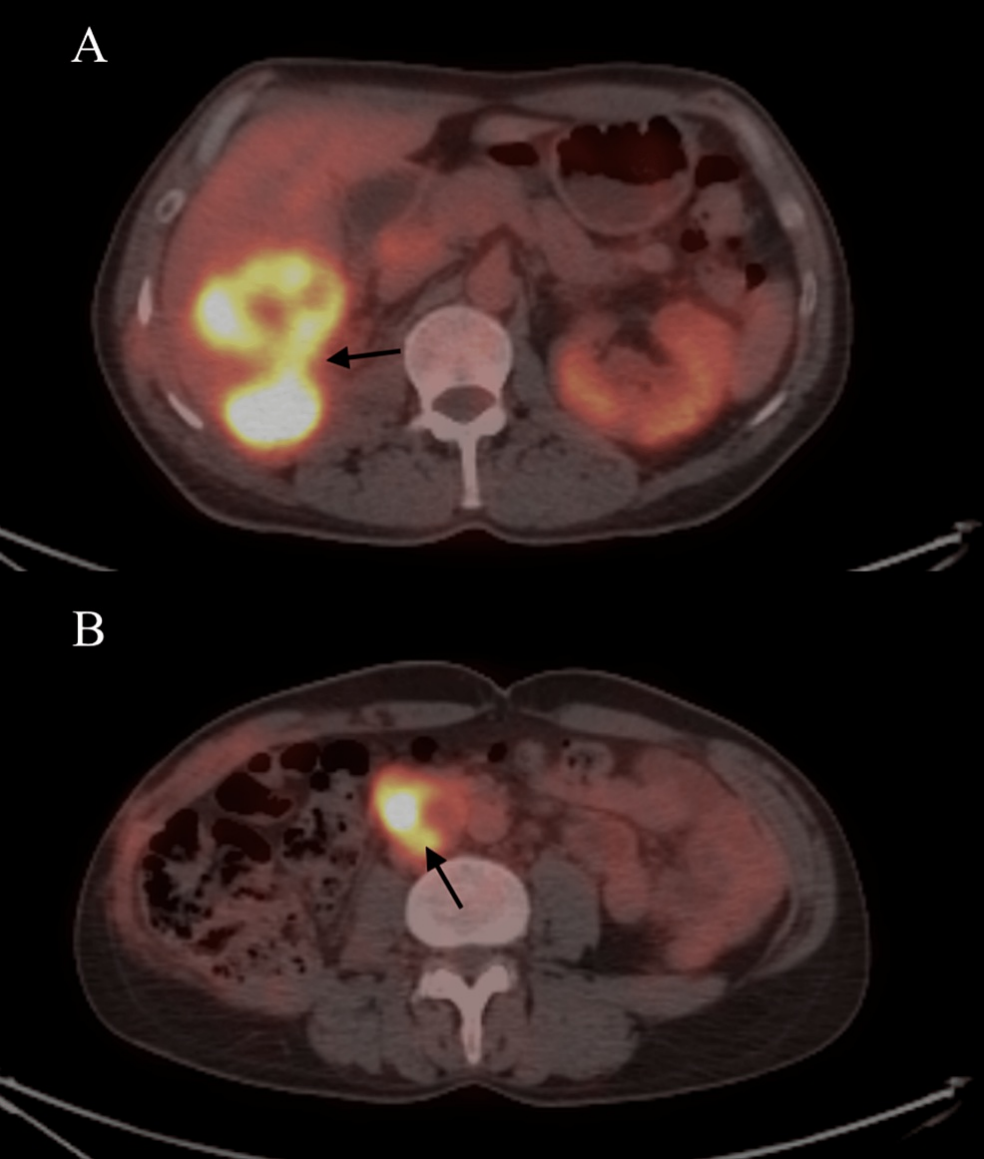
FDG-PET CT sections during follow-ups showing a metastatic mass in the liver (A) and metastatic lymph nodes in the aortocaval region (B) with arrows.

In addition, the pathological evaluation revealed that the tumor was locally invasive to the liver (pT4), there was no metastasis in the lymph nodes, and the surgical margins were negative. We referred the patient to the medical oncology department for further treatment. A non-contrast abdominal CT, which was performed before the patient received chemotherapy, showed hypodense and heterogeneous areas in the liver. The timeframe between the preoperative CT and the CT before commencing chemotherapy was 10 weeks. He was administered gemcitabine 2,000 mg/m2 along with granulocyte-colony-stimulating factor support every three weeks for three cycles. After chemotherapy, at the sixth month postoperatively, F18-fluorodeoxyglucose positron emission tomography-computed tomography (FDG-PET CT) was performed, and it showed local recurrence, a 6 cm metastatic mass in the liver (Figure 3A), and metastatic lymph nodes in the aortocaval region of 4 cm in size (Figure 3B).

The standardized uptake value (SUVmax) was measured at 15 for liver metastasis and 22.6 for lymph nodes. Despite chemotherapy and palliative treatment in the medical oncology department, the patient ultimately died of metastatic disease 11 months after surgery.

## Discussion

The fistula between the kidney and the skin is called a nephrocutaneous fistula. Acquired nephrocutaneous fistula is a rare pathology [5]. Nephrocutaneous fistulas are generally seen in patients with a history of renal surgery, trauma, tumors, and chronic urinary tract infections caused by renal stone disease, and nearly all published cases are related to chronic urinary tract infection and nephrolithiasis [6]. The presented case had a history of right-sided PCNL, and when the patient’s history and past CTs were evaluated, no evidence of kidney cancer was found. As we were investigating the patient for renal stone disease due to the presenting symptoms, we incidentally found a renal tumor in the right kidney. Considering that the patient did not have a history of kidney cancer at the time he underwent PCNL and the duration of symptoms related to nephrocutaneous fistula, we considered that the existing nephrocutaneous fistula was caused by kidney stones and urinary infection rather than by tumoral origin. However, regarding the possibility that the fistula tract is associated with the tumor, fistulectomy was performed simultaneously with radical nephrectomy in this case, and pathology revealed cancerous findings at the inner end of the fistula.

Carcinosarcoma in the urinary system generally arises in the bladder and seldom originates from the renal pelvis or ureter [7]. The most common cancer seen in the renal pelvis is TCC [8]. The first CSRP case was published by Fauci et al. in 1961 [9]. In the literature, there are mostly case reports of CSRP. The main reason for the lack of comprehensive studies on this subject is the rarity of CSRP.

In the study by Wang et al., the mean age of the patients was 72, and the patients ranged in age from 40 to 97 years old [10]. The most common symptom of CSRP is macroscopic hematuria, followed by pain; it may also cause a palpable mass [4]. The symptoms mimic upper tract TCC. Mantica et al. reported that all the cases in their study were symptomatic [4]. The most common comorbidity accompanying CSRP is nephrolithiasis. However, stone disease and chronic inflammation secondary to urinary infection usually lead to SCC [4,8]. It should be kept in mind that kidney stone disease, nephrocutaneous fistulas, and renal masses may exist concurrently. We find no case in the literature in which nephrocutaneous fistula and CSRP coexist. Although researchers suggest that the possibility of nephrocutaneous fistula being associated with stone disease and chronic infections is higher than the possibility of its being associated with CSRP, the limited data on CSRP leaves us doubtful. Renal masses should be included in the differential diagnosis in such cases due to the similarity of symptoms and the possibility that both pathologies may coexist.

Radiologic discrimination against CSRP is challenging. The literature does not provide adequate information about the imaging findings of CSRP [7]. It may resemble images of epithelial malignancies of urothelial organs, and a definitive diagnosis requires histopathological evaluation [7]. Ultrasonography and CT may be used to detect concomitant masses in the ureter and bladder [4].

Chondrosarcoma, osteosarcoma, rhabdomyosarcoma, liposarcoma, or fibrosarcoma can be observed as a mesenchymal component, and osteosarcoma or chondrosarcoma is usually encountered [3,11]. Mantica et al. reported in a systematic review that more than one epithelial or mesenchymal pathological pattern is often seen concurrently [4]. In the presented case, TCC, SCC, osteosarcoma, and chondrosarcoma are concomitant.

CSRP tends to be bulky, invasive, and fast-growing [12]. It is a high-grade tumor, has a poor prognosis, and has a low survival rate [1,8]. In a study published by Wang et al., most of the patients presented with a high histological grade and advanced-stage disease [10]. Researchers have given different data on median cancer-specific survival. Wang et al. reported the median cancer-specific survival time of CSRP as six months [10]. Dong et al. reported the median cancer-specific survival time as one year [1]. The longest survival time in the literature was reported as two years by Chen et al. [13]. Reports in the literature about the aggressiveness of CSRP make clear the importance of early diagnosis [10].

At present, there is no optimal strategy for treatment because there have been too few clinical trials. Although radical nephrectomy, chemotherapy, and radiotherapy can be applied singly or in combination, recent information suggests that among these alternatives, only radical nephrectomy is curative [1]. Cancer-specific survival time is more significantly improved by radical nephrectomy than by chemotherapy or radiotherapy [1]. Although radical resection surgery is often recommended, local recurrence, distant metastasis, or both are commonly observed in its aftermath [14]. Adjuvant chemotherapy or radiotherapy is also recommended. But postoperative chemotherapy, radiotherapy, or both combined do not contribute to survival [11,15]. There is no standard chemotherapy or radiotherapy regimen for CSRP, but some cases have responded to cisplatin and gemcitabine [16]. In the presented case, the patient received gemcitabine, but local recurrence and liver metastasis eventually occurred anyway. Local recurrence is not uncommon after radical resection [17].

Working with limited data about the histopathologic features of the metastases, Cuadra-Urteaga et al. reported that CSRP metastases usually exhibit mixed histology with a predominance of epithelial components [14,17]. Yoshida et al. reported liver, lung, and bone metastases in their case [18]. The local recurrence and liver metastasis in the presented case confirm what is reported in the literature.

## Conclusions

Carcinosarcoma of the renal pelvis is a rare biphasic tumor with both epithelial and mesenchymal components that progresses rapidly and results in death. Currently, the only known curative treatment is radical nephrectomy. Early local recurrence and the high metastatic potential of the disease cause high mortality. Since this is a rare disease, there are few reported cases. The rarity of the disease is the main obstacle to conducting comprehensive clinical trials. It is of great importance to publish the identified CSRP cases.
